# P-1280. Vaccinating Your Child During an Emergency Is More Important Than Ever: A Randomized Controlled Trial on Message Framing Among Ukrainian Refugees in Poland

**DOI:** 10.1093/ofid/ofae631.1461

**Published:** 2025-01-29

**Authors:** Dorota Kleszczewska, Agnieszka Sochoń-Latuszek, Maike Winters, Katarzyna Lewtak, Anastasiia Nurzhynska, Kseniia Yoruk, Katarzyna Kukuła, Mutribjon Bahruddinov, Aleksandra Kusek, Anna Dzielska, Tomasz Maciejewski, Joanna Mazur, Hannah Melchinger, John Kinsman, Sarah Christie, Saad Omer

**Affiliations:** Institute of Mother and Child Foundation, Warsaw, Poland, Warsaw, Mazowieckie, Poland; UNICEF Refugee Response Office in Poland, Warsaw, Poland, Warsaw, Mazowieckie, Poland; European Centre for Disease Prevention and Control, Stockholm, Stockholms Lan, Sweden; Medical University of Warsaw, Warsaw, Mazowieckie, Poland; UNICEF Eastern and Southern Africa Regional Office, Nairobi County, Nairobi Area, Kenya; UNICEF Refugee Response Office in Poland, Warsaw, Poland, Warsaw, Mazowieckie, Poland; UNICEF Refugee Response Office in Poland, Warsaw, Poland, Warsaw, Mazowieckie, Poland; UNICEF Refugee Response Office in Poland, Warsaw, Poland, Warsaw, Mazowieckie, Poland; WHO Country Office in Poland, Warsaw, Mazowieckie, Poland; Institute of Mother and Child, Warsaw, Mazowieckie, Poland; Institute of Mother and Child, Warsaw, Mazowieckie, Poland; University of Zielona Góra, Collegium Medicum, Zielona Gora, Lubuskie, Poland; Peter O'Donnell Jr. School of Public Health, UT Southwestern Medical Center, Dallas, Texas; European Centre for Disease Prevention and Control, Stockholm, Sweden, Stokholm, Stockholms Lan, Sweden; Yale School of Public Health, Yale University, Yale, Connecticut; University of Texas South Western, Dallas, Texas

## Abstract

**Background:**

Since February 2022, millions of Ukrainian women and children have fled their home country. Evidence suggests that vaccination coverage among Ukrainian refugee children is sub-optimal. We therefore tested the effect of three messages on mothers’ intention to vaccinate their children, and on scheduling a vaccination appointment.

**Methods:**

The UNICEF-funded project (SBC office) 'Say Yes to Vaccinations’ conducted an online randomized controlled trial among Ukrainian refugee mothers residing in Poland who had at least one child under 7 years of age. Participants were randomized to 1 of 3 intervention groups or to the control group. Intervention messages encouraged vaccination with a specific frame: 1) trust in the Polish healthcare system, 2) ease of access to vaccination, 3) risk aversion (highlighting the vulnerability to diseases due to emergency). Primary outcomes included intention to get a child vaccinated in Poland and clicking on a link to schedule a vaccination appointment.

**Results:**

3,282 women entered the survey, 1,233 did not meet the inclusion criteria. A total of 1,910 Ukrainian mothers completed the study, 33% had fully vaccinated children. The mothers who read the risk aversion message were significantly more likely to intend to get their children vaccinated in Poland (AOR 2.35; 95% CI 1.25-4.42). 31% of the participants in the risk aversion intervention groups clicked on the link to make the appointment compared with 25% in the control group, which translated into an AOR of 1.53 (95% CI 1.12-2.09). The messages focused on trust and ease of access did not result in increased vaccination intention or clicking on the link. Getting vaccination information from social media was associated with increased vaccination intention (AOR 1.91; 95% CI 1.21-3.00), not trusting anyone for vaccination information, however, was associated with decreased vaccination intentions (AOR 0.34; 95% CI 0.11-1.00).

**Conclusion:**

We found that framing a pro-vaccination message around risk aversion resulted in increased intention to vaccinate and more clicks on a vaccination scheduler. Health workers who interact with Ukrainian refugees could use the risk aversion frame when communicating around vaccination, highlighting the vulnerable situation faced by refugees.

**Disclosures:**

**Saad Omer, MBBS MPH PhD**, Meta: Advisor/Consultant|Meta: Grant/Research Support

